# Effects of saturation and contrast polarity on the figure-ground organization of color on gray

**DOI:** 10.3389/fpsyg.2014.01136

**Published:** 2014-10-07

**Authors:** Birgitta Dresp-Langley, Adam Reeves

**Affiliations:** ^1^ICube UMR 7357 CNRS/Université de StrasbourgStrasbourg, France; ^2^Psychology Department, Northeastern UniversityBoston, MA, USA

**Keywords:** color perception, luminance contrast sensitivity, depth perception, achromatic contrast discrimination, colorimetry

## Abstract

Poorly saturated colors are closer to a pure gray than strongly saturated ones and, therefore, appear less “colorful.”Color saturation is effectively manipulated in the visual arts for balancing conflicting sensations and moods and for inducing the perception of relative distance in the pictorial plane. While perceptual science has proven quite clearly that the luminance contrast of any hue acts as a self-sufficient cue to relative depth in visual images, the role of color saturation in such figure-ground organization has remained unclear. We presented configurations of colored inducers on gray “test” backgrounds to human observers. Luminance and saturation of the inducers was uniform on each trial, but varied across trials. We ran two separate experimental tasks. In the relative background brightness task, perceptual judgments indicated whether the apparent brightness of the gray test background contrasted with, assimilated to, or appeared equal (no effect) to that of a comparison background with the same luminance contrast. Contrast polarity and its interaction with color saturation affected response proportions for contrast, assimilation and no effect. In the figure-ground task, perceptual judgments indicated whether the inducers appeared to lie in front of, behind, or in the same depth with the background. Strongly saturated inducers produced significantly larger proportions of foreground effects indicating that these inducers stand out as figure against the background. Weakly saturated inducers produced significantly larger proportions of background effects, indicating that these inducers are perceived as lying behind the backgrounds. We infer that color saturation modulates figure-ground organization, both directly by determining relative inducer depth, and indirectly, and in interaction with contrast polarity, by affecting apparent background brightness. The results point toward a hitherto undocumented functional role of color saturation in the genesis of form, and in particular figure-ground percepts in the absence of chromatostereopsis.

## Introduction

**Figure 1:**
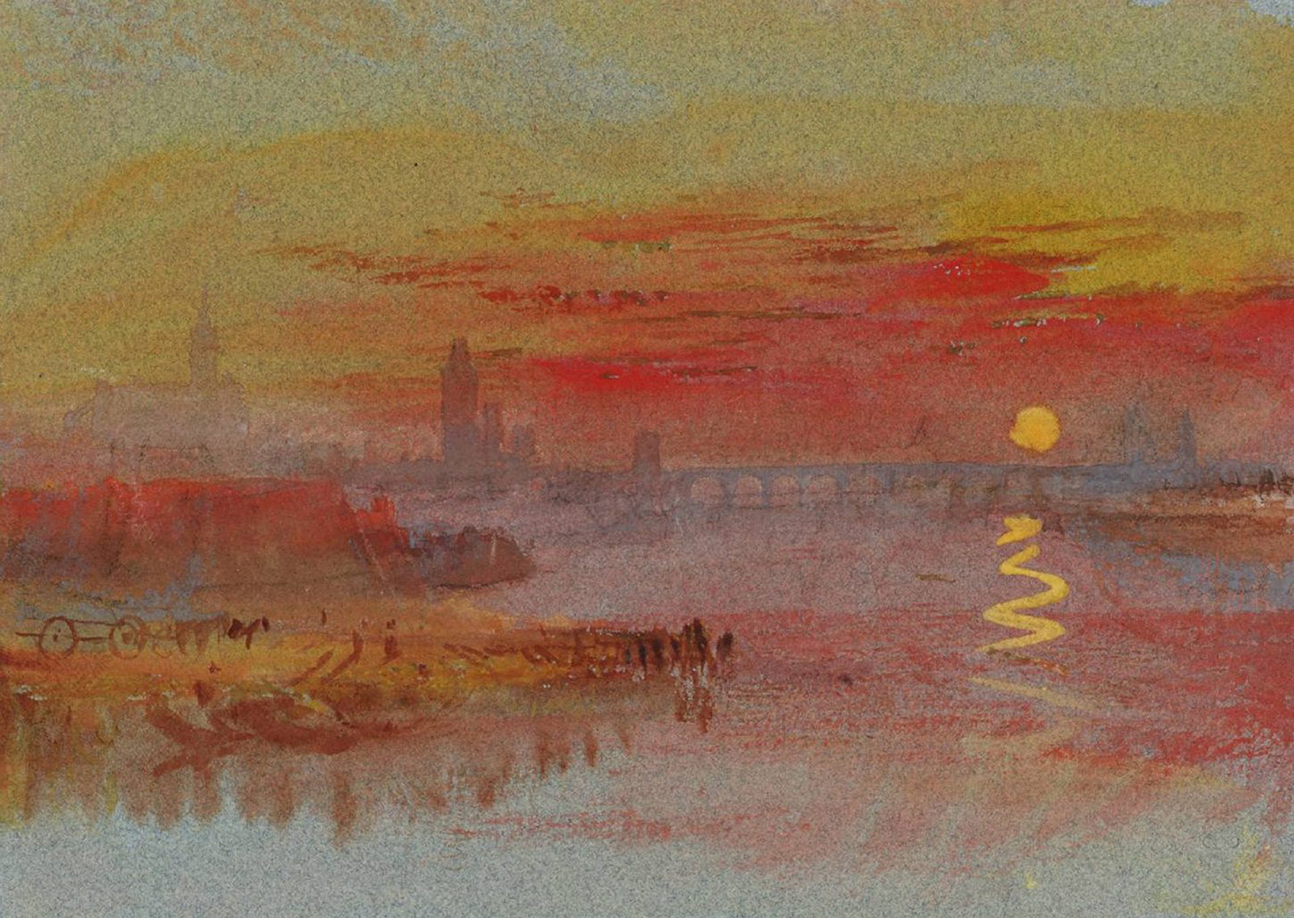
**In the nineteenth century, painters like Turner effectively exploited color, saturation, and luminance effects to suggest figure and ground, as here in “Sunset on Rouen”**.

**Figure 2:**
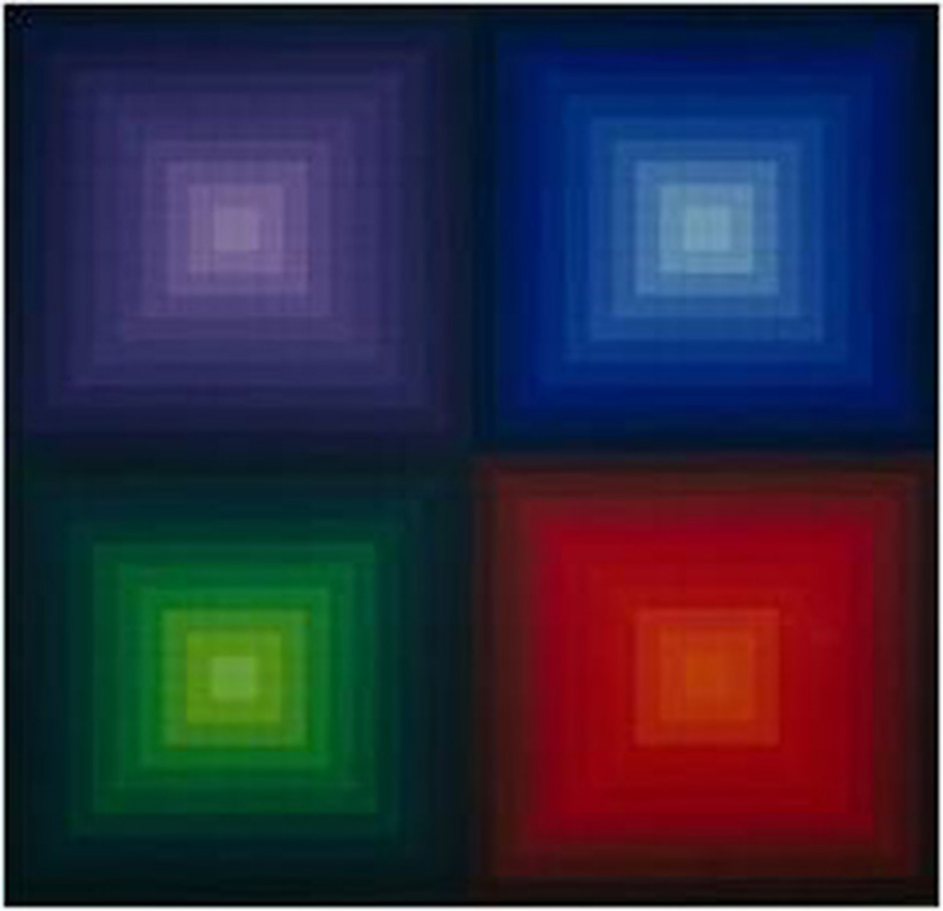
**In the twentieth century, designers like Vasarély, as here in “Arcturus II,” demonstrated how the manipulation of color, saturation, and luminance contrast cooperates with planar shape geometries for the generation of compelling figure-ground effects**.

Poorly saturated colors, since they are closer to a pure gray than intense hues, appear less “colorful” than strongly saturated colors, yet, they still contain hue information. In the visual arts, color saturation is widely exploited as a measure for balancing opponent or conflicting sensations and moods. In the nineteenth century, at the dawn of abstract expressionism, painters such as Turner (especially in his later works) effectively used color to suggest what should be nearer or further away to the observer in the painting, relying on chromatic brightness, and saturation to express and balance figure and ground, moods, and other qualia (Figure 1). The earlier Renaissance painters had preferentially resorted to chiaroscuro and geometric cues to aerial perspective using a limited chromatic range to create landscape depth and figure-ground effects. Later in the evolution of visual art, modern architects and designers like Vasarély effectively manipulated color saturation in combination with planar shape geometry to play with foreground and background effects in a complex and abstract manner (Figure 2), illustrating how chromatic luminance, saturation, and shape can be combined to elicit powerful visual sensations suggesting three-dimensional structure. While contemporary visual artists tend to share the strong belief that saturation is a key medium for creating perceptual structure, perceptual science has not yet clarified the functional contribution of color saturation to perceptual organization. Imagine the simplest possible two-dimensional image with no more than two adjacent surface regions. When there is a difference in brightness between the two adjacent regions, they can constitute a figure-ground reversible pattern, where the region seen as figure is perceived in front of the region seen as ground. This difference in perceived depth between the two regions increases as their difference in brightness increases. The observation originally stems from an experiment by Egusa (1977), who presented two different achromatic surfaces, viewed through a small aperture, on a black screen. The surface on the right was of one of three different shades of gray, and the one on the left was either white or black. Observers made judgments regarding the apparent depth of these surfaces in terms of which of the two appeared nearer. The results of this study were the first to reveal a systematic relation between perceived relative depth and brightness differences between adjacent surface regions, in that increasing the brightness difference increased the perceived depth separation in every observer. Whether the brighter or the darker of the two test surfaces appeared nearer differed from observer to observer. Subsequently, Egusa (1983) examined the effects of brightness, hue, and saturation on the perceived depth between two adjacent regions. Again, the stimuli consisted of two hemifields, either both achromatic, one achromatic the other chromatic, or of two different colors. Subjects were asked to state which hemifield appeared nearer, and to put a number on the perceived depth between (depth magnitude estimation). When both hemifields were achromatic, the perceived depth was found to increase with increasing brightness difference. Again, some subjects tended to judge the brighter side nearer, others the darker side. With the achromatic-chromatic combination, there were no differences in perceived depth among three hue conditions, whilst with the chromatic-chromatic combination the perceived depth depended on the hue combination. In terms of decreasing frequency of “nearer” judgments, the hue order was red, green, and blue. When two chromatic hemifields differed in saturation only, the perceived depth increased with increasing difference in saturation, and whether the more saturated or the less saturated side was judged nearer depended on hue. Thus, the figure-ground differentiation between two adjacent chromatic regions in the visual field is jointly determined by brightness, hue and saturation, affecting the perceived distance of a given region from the observer.

Since Leonardo Da Vinci's *Trattato della Pittura* (Da Vinci, 1651) mentioning luminance contrast as a cue to pictorial depth, perceptual science has confirmed that it directly determines what will be seen as nearer or further away in two-dimensional visual configurations and images (Mount et al., 1956; Farnè, 1977; Rohaly and Wilson, 1993; O'Shea et al., 1994; Dresp et al., 2002; Guibal and Dresp, 2004; Dresp-Langley and Reeves, 2012). These observations, however, do not cover possible interactions between hue, luminance contrast, and saturation on the figure-ground organization of color adjacent to, or surrounded by, achromatic fields, or gray tones of varying luminance intensity. Reasons why such effects were not actively searched for may relate to the fact that chromatic and achromatic pathways in the visual brain are widely believed to be independent (e.g., Page and Crognale, 2005), presuming no functional interaction between chromatic and achromatic neural signals.

To clarify whether or not saturation influences figure-ground perception of color patterns on achromatic backgrounds, we used stimuli from a previous study (Dresp-Langley and Reeves, 2012), which had already shown that colors of any hue could alter the perceived intensity of their achromatic backgrounds, pointing toward hitherto unsuspected interactions between color signals and achromatic contrast signals. Also, colors on gray produce depth effects that can directly be explained by variations in their luminance contrast irrespective of hue. Here in this study, we varied the saturation levels, luminance contrast, and contrast polarity of colored and/or achromatic inducers on gray backgrounds in planar configurations that may be similar to complex patterns in natural images. We tested test how these variations affect perceived background brightness and figure-ground organization. In a relative background brightness task, observers were asked to indicate whether a gray background containing colored or achromatic inducers appeared “brighter” than, “darker” than, or the “same” as a comparison background, which contained no inducers. In a relative depth (figure-ground) task, observers were asked to indicate whether colored, or achromatic inducers appeared to stand “in front of” or “behind” their gray background surface, or whether all surfaces appeared to lie in the “same” plane.

## Materials and methods

Experiments were run under Windows XP on a Dell PC computer equipped with a mouse device and a high resolution color monitor (EIZO LCD “Color Edge CG275W”) with an in-built color calibration device (colorimeter), which uses the Color Navigator 5.4.5 interface for Windows. The colors of the stimuli were generated in Photoshop using selective combinations of Adobe RGB increments. The color coordinates (see Table 1) for each RGB triple are retrieved from the look-up table of the colorimeter after calibration. All luminance values for calculating the stimulus contrasts (see the Michelson contrasts, with negative and positive contrast polarities, here in Table 2) were determined on the basis of standard photometry using an external photometer and adequate interface software (Cambridge Research Instruments).

**Table 1:** **Color coordinates (X, Y, Z) and RGB (Adobe) triplets associated with the different hues (shown on their gray backgrounds here in Figure 3)**.

	**Color coordinates**							
	**“Weakly” saturated hues**				**“Fully" saturated hues**			
	***x***	***y***	***z***	**RGB**	***x***	***y***	***z***	**RGB**
**COLOR APPEARANCE**								
≪Light≫ RED	0.33	0.33	0.34	[235, 197, 197]	0.68	0.31	0.01	[250, 0, 0]
≪Dark≫ RED	0.33	0.33	0.34	[127, 99, 99]	0.68	0.31	0.01	[100, 0, 0]
≪Light≫ GREEN	0.31	0.35	0.34	[183, 221, 183]	0.20	0.70	0.10	[0, 250, 0]
≪Dark≫ GREEN	0.31	0.35	0.34	[91, 110, 91]	0.20	0.70	0.10	[0, 100, 0]
≪Light≫ BLUE	0.29	0.30	0.41	[180, 201, 255]	0.15	0.05	0.80	[0, 0, 150]
≪Dark≫ BLUE	0.29	0.30	0.41	[90, 104, 160]	0.15	0.05	0.80	[0, 0, 125]
≪Light≫ YELLOW	0.32	0.36	0.32	[220, 220, 175]	0.42	0.51	0.07	[255, 255, 0]
≪Dark≫ YELLOW	0.32	0.36	0.32	[130, 123, 85]	0.42	0.51	0.07	[100, 100, 0]

**Table 2:** **Michelson contrasts (four per hue and saturation level and four additional achromatic conditions) of the inducer-background configurations**.

**Hues**	**“Fully” saturated**				**“Weakly” saturated**			
RED inducers	−0.44	−0.08	0.58	0.79	−0.21	0.30	0.73	0.91
GREEN inducers	−0.57	0.35	0.47	0.91	−0.38	0.35	0.63	0.91
BLUE inducers	−0.90	−0.67	−0.29	0.32	−0.38	0.14	0.63	0.86
YELLOW inducers	−0.78	0.11	0.51	0.94	−0.36	0.30	0.65	0.92
ACHROMATIC inducers	−0.45	0.52	0.59	0.94				

### Subjects

Ten unpracticed observers, mostly graduate students in computational and/or design engineering and unaware of the hypotheses of the study, participated in the experiments. We obtained informed consent from all of them, in compliance with international ethical standards for experimentation on human observers. All subjects had normal or corrected-to-normal visual acuity and normal color vision (assessed on the basis of the Ishihara plates).

### Stimuli

**Figure 3:**
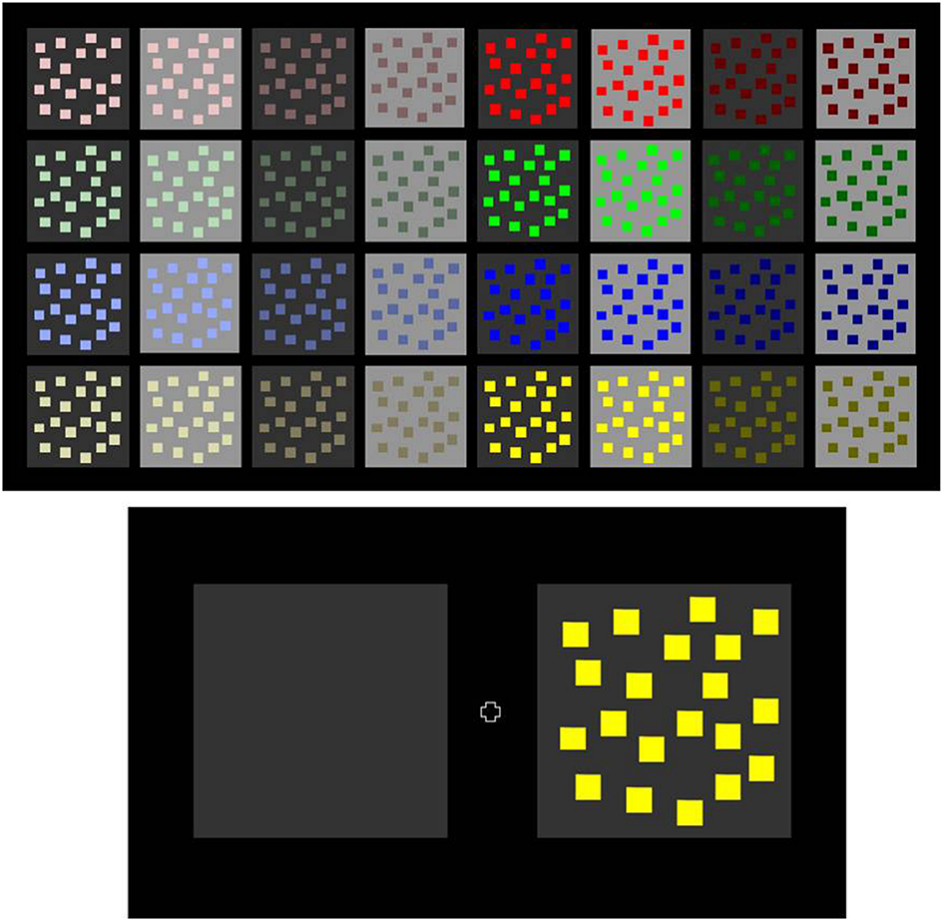
**We varied the luminance contrast and saturation (as shown in the upper panel here) of colored inducers presented on gray backgrounds**. In the relative background brightness task, observers had to judge the relative brightness (brighter, darker, or same) of a gray test background with inducers in comparison with a simultaneously presented background field, of identical luminance but without inducers (as shown in the lower panel here). In this pair of configurations, 90% of subjects perceive the gray background with inducers on the right as darker than the plain comparison background on the left, a typical subjective contrast effect. In the relative inducer depth task, observers had to indicate the relative depth of the inducers in *single* inducer-background configurations (there was no comparison field). They had to decide whether inducers are seen as standing in front or behind the gray background, or whether inducers, and background appeared to lie in the same plane. In the configuration shown on the right of the lower panel here, 95% of subjects perceive the colored inducers as standing in front of their gray background, a typical foreground effect.

The stimuli (see Figure 3 upper panel) consisted of configurations of 20 colored square-shaped surfaces, as from now called inducers, placed on a gray square-shaped surface which formed the surrounding background, and displayed on a black (0 cd/m^2^) computer screen. The hue of the inducers could be red, green, blue, yellow, or achromatic (gray). The saturation of the inducer colors was varied to produce configurations with fully saturated and configurations with weakly saturated hues (see Table 1). Inducer luminance (in cd/m^2^) was 22.1 and 9.9 for strongly saturated red, 53.2 and 16.7 for weakly saturated red; 54.0 and 7.1 for strongly saturated green, 53.9 and 11.5 for weakly saturated green; 5.1 and 1.4 for strongly saturated blue, 34.6 and 11.6 for weakly saturated blue; 79.0 and 3.2 for strongly saturated yellow, 12.3, 58.8, and 12.3 for weakly saturated yellow. The luminance of achromatic inducers was 9.95 and 82.70 cd/m^2^. The luminance of the gray backgrounds was 2.6 and 25 cd/m^2^. Inducer-background combinations produced eight contrast levels, one for each hue of a given saturation on each background, four per saturation level of a given hue, and four contrast levels for achromatic combinations. These Michelson contrasts, calculated on the basis of (L_max_ − L_min_)/(L_max_ + L_min_), are given in Table 2. There was at least one negative contrast polarity for each level of hue and saturation. Color coordinates (X, Y, Z) for inducer colors are given in Table 1 as a function of color appearance and the two saturation levels. In the task where observers had to judge relative background brightness, two background configurations were presented simultaneously: a configuration with inducers on the test background, and a comparison background without inducers (see lower panel in Figure 3). The location of test and comparison backgrounds on the screen varied randomly between left and right. A small fixation cross of low intensity was presented between trials to help subjects fixate the center of the screen. The horizontal distance between two backgrounds on the screen was 4 cm, and a given configuration on each side was 1.5 cm away from the central fixation mark that appeared between trials. The height of each background square was 9.7 cm and the width 10 cm. The smallest horizontal distance between colored inducers was 0.4 cm, the smallest vertical distance 0.5 cm. All colored inducers had identical height (0.9 cm) and width (1 cm). In the task where observers had to judge relative inducer depth, a single inducer-background configuration was displayed centrally on the screen on each trial.

### Task instructions

Both experimental tasks used three-alternative forced choice to measure perceptual decisions. In the background contrast task, observers were asked to indicate whether the gray background containing inducers appeared “brighter” than, “darker” than, or the “same” as the comparison background, which contained no inducers. It was made clear that all subjects had understood that they were to compare the relative brightness of the two gray backgrounds on either side of the screen. In the relative depth or figure-ground task, observers were asked to indicate whether the colored inducer surfaces appeared to stand “in front of” or “behind” their gray background surface, or whether all surfaces appeared to lie in the “same” plane. It was made sure that all observers understood the instructions correctly before an experiment was initiated.

### Procedure

Subjects were seated at a distance of 1.5 m from the screen, their heads comfortably resting on a head-and-chin support. The experiments were run in a dimmed room, with blinds closed on all windows (mesopic range). Previous research had established that rod vision is not required for the generation of either apparent brightness or depth with the type of stimuli used here (Dresp-Langley and Reeves, 2012). Five of the ten observers were run in the relative background brightness task first and then in the relative depth task, the other five were run in reverse order. In the task where observers had to judge relative background brightness, two background configurations were presented simultaneously, a test background with inducers on one side of the screen (randomly on the left or right) and a comparison background without inducers on the other side. In the task where observers had to judge relative inducer depth, a single inducer-background configuration was displayed centrally on the screen on each trial. In each task or session, the configurations were presented in random order for about 1 s each and each configuration was presented twice. Inter-stimulus intervals typically varied from 1 to 3 s and were placed under the control of the subject to allow for any after-images to vanish before the next trial was initiated. Between stimuli, subjects were exposed to a uniformly black screen, with a small, slightly brighter, fixation cross displayed in the center, which was to help them control the direction of gaze. Each individual session consisted of 72 trials per subject, of which 64 with colored inducers on the gray backgrounds and eight with achromatic inducers on the gray backgrounds.

## Results and discussion

The data from each task were analyzed separately. Response proportions were determined on the basis of the frequency with which a given effect was observed in each of the two tasks. Relative background brightness (task 1) was assessed on the basis of frequencies of contrast effects, assimilation effects, and responses signaling no effect. Relative inducer depth or figure-ground (task 2) was assessed on the basis of frequencies of foreground effects (“in front”), background effects (“behind”), and responses signaling no effect.

### Contrast and assimilation of the gray backgrounds

We determined frequencies (F) of contrast effects reflecting responses where a test background containing brighter inducers was judged “darker” than the comparison field, or a test background containing darker inducers was judged “brighter” than the comparison field. Frequencies of assimilation effects reflect responses where a test background containing brighter inducers was judged “brighter” than the comparison field, or where a test background containing darker inducers was judged “darker” than the comparison field. Frequencies of no effect reflect responses where the test background appeared of the same brightness as the comparison field (Experiment 1). These frequencies were then transformed into response proportions *P* = *F/N* where *N* is the number of observations in a given condition.

Since inter-individual differences are not uncommon in this type of task (Egusa, 1977, 1983), we checked the raw data in the spreadsheet first for inter-observer consistency, which was good (10 two-by-two comparisons returned 80–85% matches between observers). Several statistical analyses were then performed on average values for *P* per experimental condition using a recent version of *Systat*, which systematically checks that conditions of normality and equality of variance are satisfied before generating further output. All comparisons given here below (analyses 1–4) had passed both tests.

Analysis 1: Two-Way ANOVA for a 2 × 4 factorial design was performed first, with two levels of the saturation factor (weak saturation, strong saturation) and the four levels of the hue factor (red, green, blue, yellow). The results of this first analysis signaled no statistically significant effects of hue or saturation on the response proportions for contrast, assimilation or, redundantly, no effect. For the next analysis (analysis 2), the four different hues were grouped with regard to luminance (Michelson) contrast and split into two polarity groups. The eight strongest positive contrasts (shown in Table 2 on the right of each panel of values for a given saturation level) formed one group, and the remaining eight (on the left of each panel for a given saturation level in Table 2), of which most were negative, the second group.

Analysis 2: A Two-Way ANOVA for a 2 × 2 factorial design was performed, with the two levels of saturation and the two levels of contrast polarity, as defined here above. These statistics signaled significant effects of contrast polarity on response proportions for contrast [*F*_(1, 1)_ = 39.36, *p* < 0.001], assimilation [*F*_(1, 1)_ = 66.83, *p* < 0.001], and no effect [*F*_(1, 1)_ = 22.20, *p* < 0.001], and statistically significant interactions between saturation, and contrast polarity on these response proportions [*F*_(1, 1)_ = 6.18, *p* < 0.05 for contrast, *F*_(1, 1)_ = 8.37, *p* < 0.01 for assimilation, and *F*_(1, 1)_ = 8.29, *p* < 0.01 for no effect].

**Figure 4:**
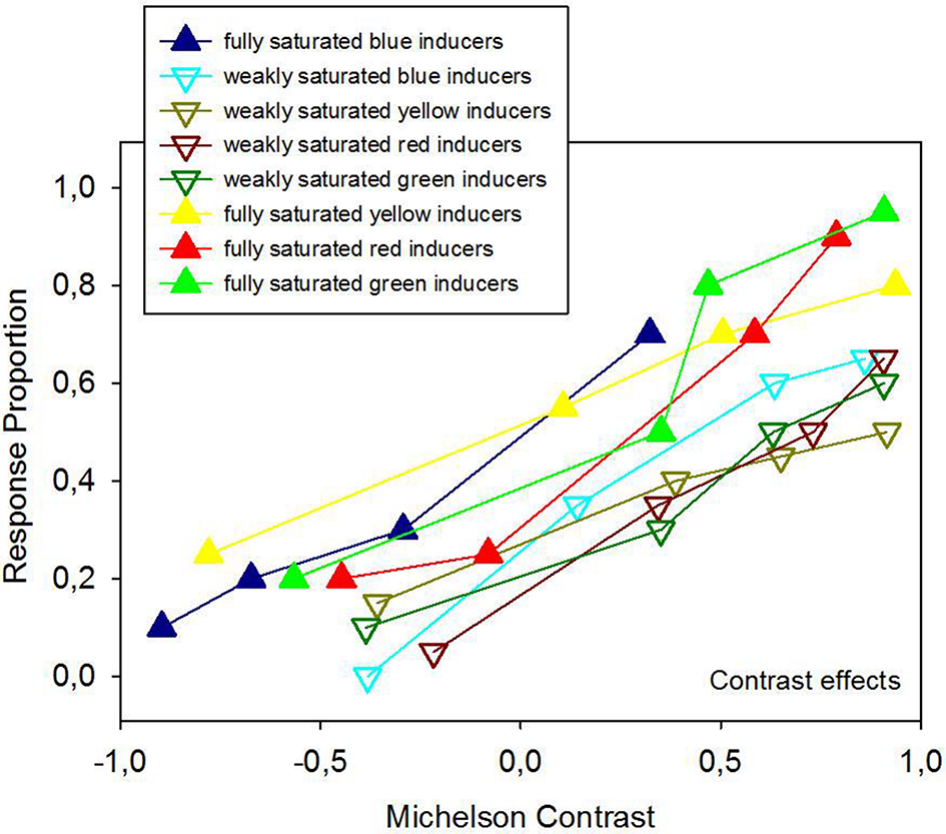
**Average response proportions for contrast effects as a function of hue, Michelson contrast, and saturation levels of the colored inducers**. Although 2 × 2 ANOVA on these means (analysis 1) did not signal statistically significant effects of hue and saturation, there is a systematic trend for fully saturated hues to produce stronger contrast effects compared with weakly saturated hues.

**Figure 5:**
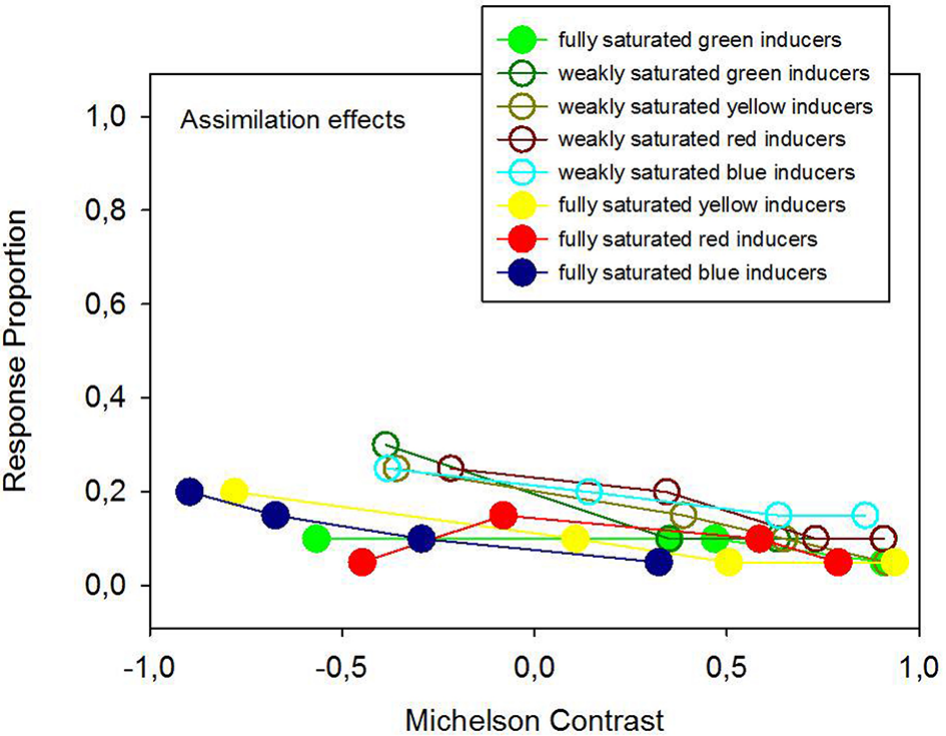
**Average response proportions for assimilation effects as a function of hue, Michelson contrast, and saturation levels of the colored inducers**. Although 2 × 2 ANOVA (analysis 1) did not signal statistically significant effects of hue and saturation, there is a systematic trend for weakly saturated hues to produce stronger assimilation effects compared with fully saturated hues.

Average response proportions (P) for contrast, assimilation, and no effect are plotted in Figures 4, 5 as a function of the hue, Michelson contrast, and saturation level of the inducers (analysis 1). The graphs show that fully saturated inducers have a tendency to yield higher proportions of background contrast than weakly saturated inducers, while weakly saturated inducers have a tendency to yield higher proportions of background assimilation than fully saturated inducers although the main effect of saturation is not statistically significant, but significantly interacts with the effect of contrast polarity. The effect of saturation depends on contrast polarity. This significant interaction between saturation and contrast polarity is reflected by several observations. Strongly saturated inducers with the highest positive luminance contrast produced the largest proportions of contrast effects, while the weakly saturated inducers with the highest negative luminance contrast produce the smallest proportion of contrast effects (Figure 4). Although, at a first glance, weakly, and strongly saturated inducers seem to produce more or less evenly distributed proportions of assimilation at all luminance contrasts, the largest proportion of assimilation effects is observed with the weakly saturated inducers of the highest negative Michelson contrast, while the smallest proportion of assimilation arises from the strongly saturated inducers with the highest positive Michelson contrast (Figure 5).

**Figure 6:**
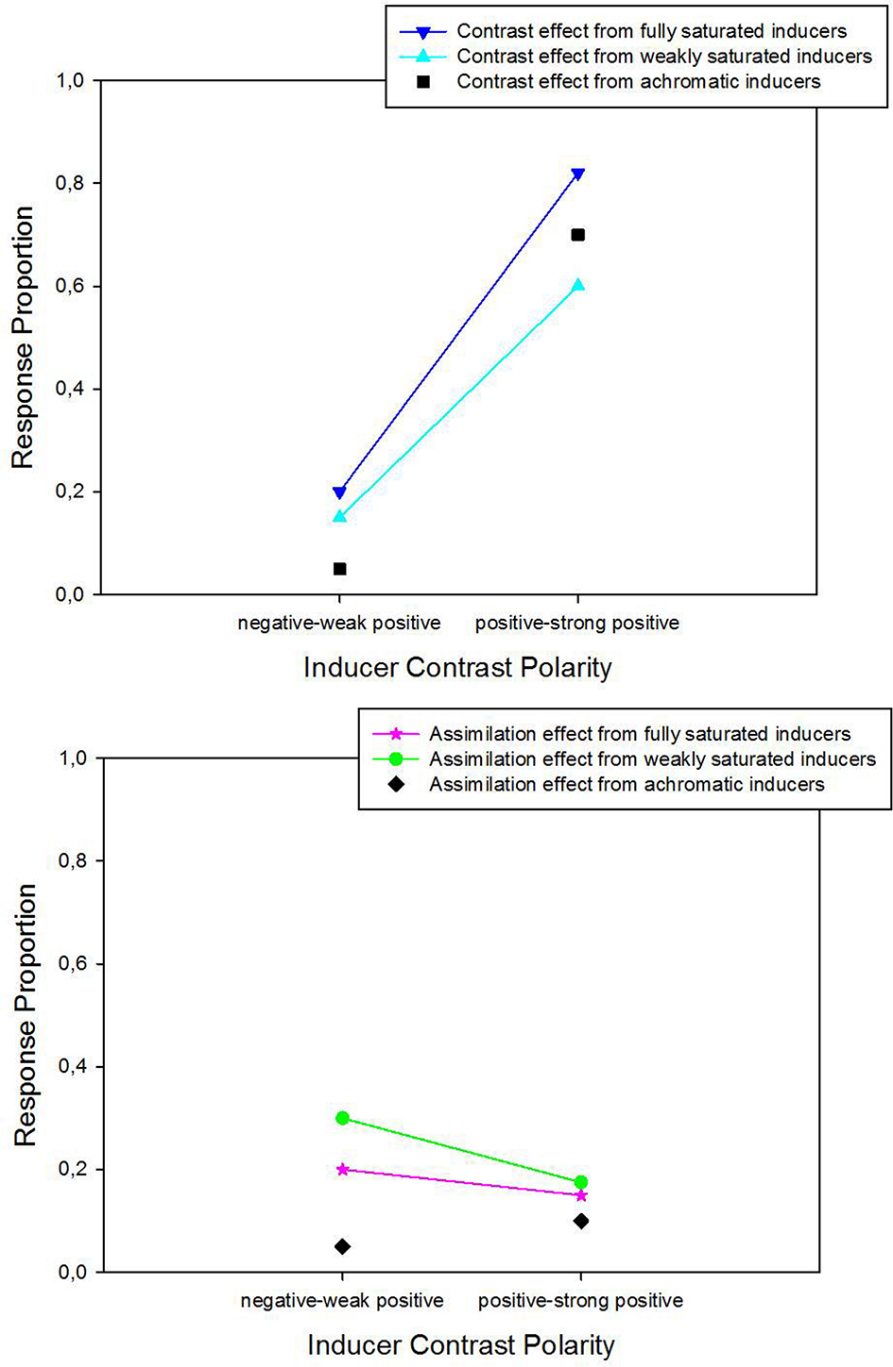
**Average response proportions for contrast (upper panel) and assimilation (lower panel) as a function of the contrast polarity and saturation levels of the colored inducers**. Data from the different hues were grouped and divided into two balanced categories (negative-to-weak-positive and positive-to-strong positive) to test for effects of contrast polarity (analysis 2). 2 × 2 ANOVA on means signaled statistically significant effects of contrast polarity and significant interactions between contrast polarity and saturation, on contrast (shown here in the upper graph) and assimilation (shown in the lower graph). Average data from the conditions with achromatic inducer-background configurations are shown for comparison.

The significant interaction between saturation and contrast polarity on the effects in this experiment is highlighted by the data show in Figure 6, which regroups the means of observations within each of the two contrast polarity categories (analysis 2). Response proportions for trials with the achromatic inducers are included in this graph for comparison. The strongest contrast effects (upper panel in Figure 6) are generated by fully saturated colored inducers with positive contrast polarity. Inducers with negative contrast sign produce very little. The assimilation effects (lower panel in Figure 6) are markedly smaller than the contrast effects. The comparison of data with colored inducers with the data from the achromatic inducers shows that all induction effects, including the achromatic ones, depend on contrast polarity. Simple explanations or models in terms of summative effects of differences in contrast, where brightness would be a fixed weighted sum of these latter (e.g., Burns et al., 1982), do not hold in the light of this dependency on contrast polarity.

### Figure-ground organization

The following analyses concern individual frequencies (F) of responses signaling figure and ground, reflecting observations where inducers were judged as standing “in front” or “behind” the gray background (Experiment 1). Frequencies of responses signaling no effect reflected individual observations where the inducers were judged to lie in the “same” plane as their gray background. The subjects' responses were analyzed for each experimental condition and individual. The responses frequencies (F) were transformed into response proportions *P* = *F*/*N* where *N* is the number of observations in a given condition.

Analysis 3: Two-Way ANOVA for a 2 × 2 factorial design with the two levels of the saturation factor and the four levels of the hue factor signaled no significant effects for hue (see also Dresp-Langley and Reeves, 2012). The effects of saturation on response proportions for foreground effects, background effects, and no effect were all statistically significant [*F*_(1, 1)_ = 7.49, *p* < 0.01 for “in front,” *F*_(1, 1)_ = 4.761, *p* < 0.05 for “behind”).

Analysis 4: ANOVA for a 2 × 2 factorial design with the two levels of the saturation factor and two levels of the polarity factor was performed. As before, the luminance contrasts associated with the four hues were split into two polarity groups according to the same principle. In addition to the significant effect of saturation (see above), this analysis revealed a significant effect of contrast polarity on the proportion of responses signaling foreground effects [*F*_(1, 1)_ = 62.20, *p* < 0.001], background effects [*F*_(1, 1)_ = 14.90, *p* < 0.01], and no effect [*F*_(1, 1)_ = 32.21, *p* < 0.001]. A significant interaction between saturation and contrast polarity was found to influence the response proportions for background effects [*F*_(1, 1)_ = 18.33, *p* < 0.01].

**Figure 7:**
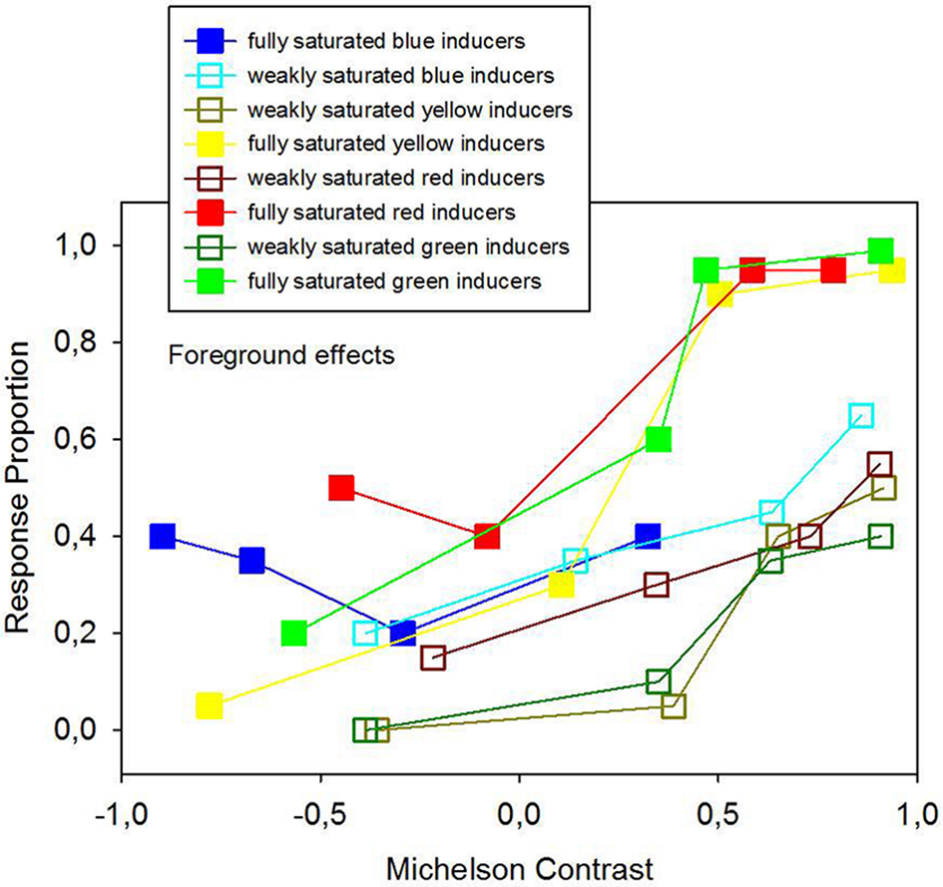
**Average response proportions of foreground effects, indicating that the colored inducers were seen as figure, as a function of hue, Michelson contrast and saturation levels of the colored inducers**. 2 × 2 ANOVA signaled a statistically significant effect of saturation (analysis 3) on foreground effects, where fully saturated inducers produce stronger figure percepts than weakly saturated inducers, as shown here in the graph.

**Figure 8:**
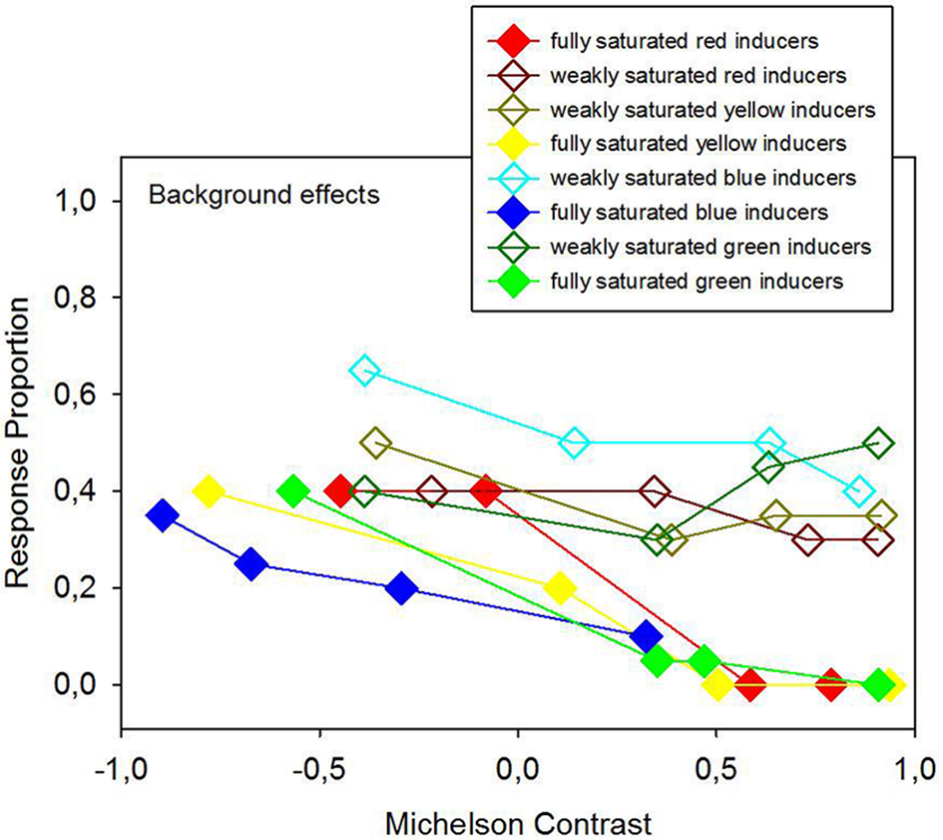
**Average response proportions of background effects, indicating that the colored inducers were seen as ground, as a function of hue, Michelson contrast, and saturation levels of the colored inducers**. 2 × 2 ANOVA signaled a statistically significant effect of saturation (analysis 3) on background effects, where weakly saturated inducers produce stronger ground percepts than fully saturated inducers, as shown here in the graph.

Average response proportions (P) for figure and ground, expressed in terms of foreground effects (“in front”) and background effects (“behind”), and response proportions relative to no effect are given in Figures 7, 8 as a function of hue, Michelson contrast, and saturation levels (analysis 3). Strongly saturated inducers produce significantly larger response proportions for foreground effects than weakly saturated inducers (Figure 7). Strongly saturated inducers with the strongest positive luminance contrasts produce the largest response proportions relative to foreground effects, where the inducers are seen as standing in front of the configuration. Weakly saturated inducers yield significantly larger response proportions for background effects than strongly saturated ones (Figure 8).

**Figure 9:**
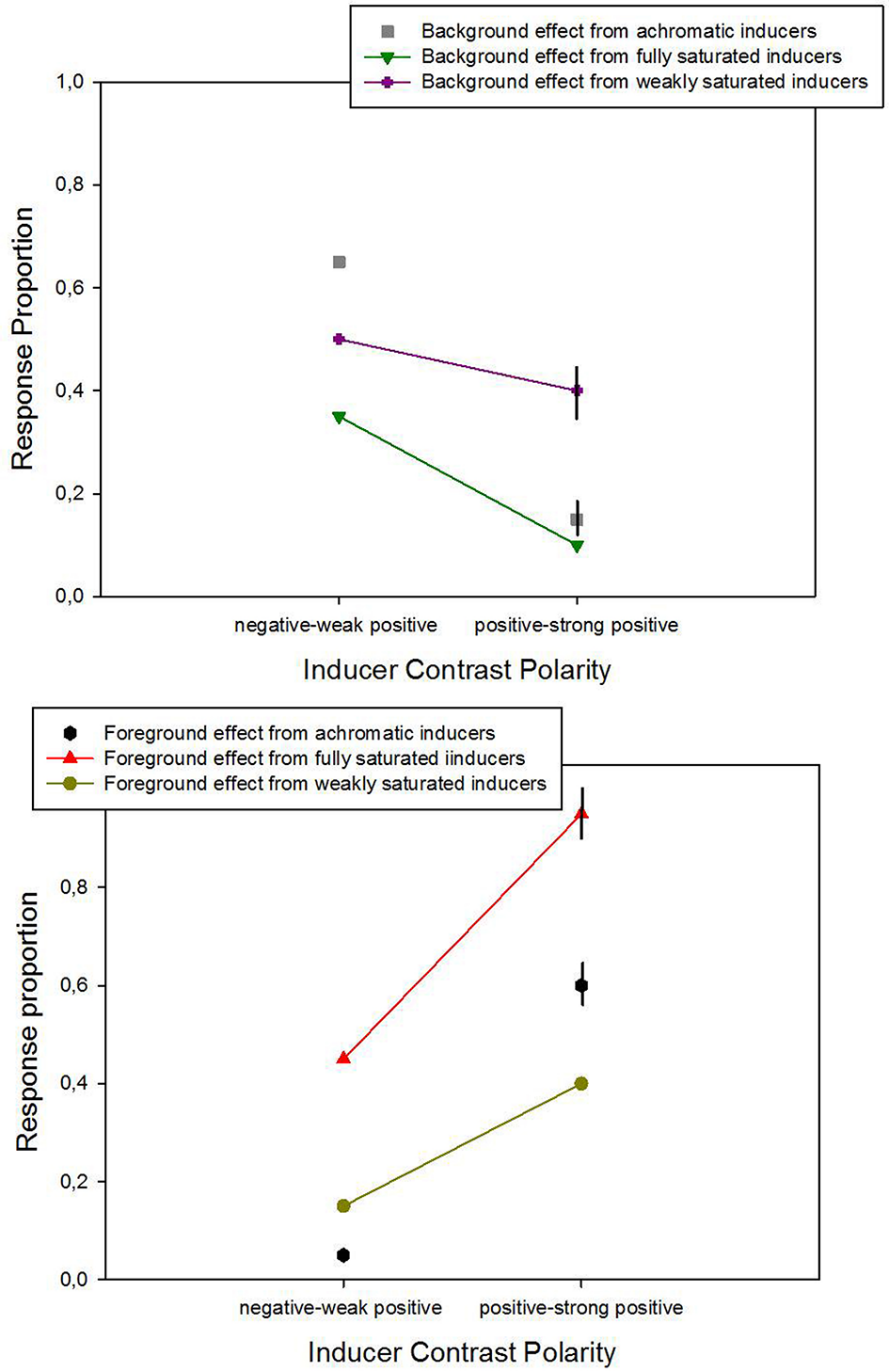
**Average response proportions for background (upper panel) and foreground effects (lower panel) as a function of the two contrast polarity categories (analysis 4)**. 2 × 2 ANOVA signaled statistically significant effects of contrast polarity on background (top) and foreground (bottom) effects. *Post-hoc* paired comparisons between individual means from achromatic inducer-background configurations and data from configurations with color on gray signaled a significant difference between achromatic and weakly saturated inducers for background effects in the positive contrast polarity range (top, with error bars for the two conditions of that comparison), and a significant difference between achromatic and fully saturated inducers for foreground effects (bottom, with error bars for the two conditions relative to that comparison).

Average response proportions (P) for figure and ground in terms of foreground and background effects are summarized as a function of the two contrast polarity categories (analysis 4) in Figure 9. Response proportions for trials with the achromatic inducers are included in this graph for comparison. Achromatic inducers with negative-to-weak positive luminance contrast and weakly saturated colored inducers with negative-to-weak-positive polarity yield the largest average response proportion for background effects (upper panel in Figure 9), while achromatic and fully saturated colored inducers with medium-to-strong positive luminance contrast yield the largest average response proportion for foreground effects lower panel in Figure 9). Proportions of foreground effects indicating that inducers are seen as figure tend to increase between negative and positive contrast polarities, while background effects indicating that inducers are seen as ground tend to decrease. The average data as plotted in Figure 9 highlight the statistically significant effects (analysis 4) of saturation and contrast polarity on the relative depth judgments (Experiment 2) even more, showing that fully saturated and weakly saturated inducers of similar luminance contrast produce markedly different effects within a given range of contrast polarities.

## Discussion

Hue as such does not significantly affect relative background brightness or figure-ground organization in displays consisting of colored inducers on gray backgrounds under the conditions tested here. This result replicates a finding from an earlier study with similar configurations and tasks (Dresp-Langley and Reeves, 2012). To examine the possibility of a contribution of hue further, it would be interesting to pursue these experiments by testing the effects of iso-luminant inducer-background configurations with different hues in both type on relative background brightness (induction effects) and relative depth judgments.

The influence of saturation on relative background brightness (induction effects) is shown here to depend on contrast polarity. The significant interaction between saturation and contrast polarity found here may be summarized as follows. Strongly saturated inducers with the medium-to-strong positive luminance contrast produced the largest proportions of contrast effects, while weakly saturated inducers with the negative luminance contrast produce the largest proportion of assimilation effects. We infer that color saturation modulates figure-ground organization indirectly, in interaction with contrast polarity, by affecting apparent background brightness.

Color saturation significantly contributes to the figure-ground organization of the colored inducers on the achromatic backgrounds. Strongly saturated surface colors associated with a positive luminance contrast are the most likely to promote foreground effects, i.e., to be seen as standing in front of their achromatic backgrounds. Weakly saturated surface colors associated with a negative luminance contrast are the most likely to generate background effects, i.e., to be seen as standing behind their achromatic backgrounds.

It has been suggested that the figure-ground organization of colored surfaces on achromatic backgrounds is more ambiguous compared with that of achromatic inducers on achromatic backgrounds. This seems to hold especially in the range of strong negative luminance contrasts, where achromatic inducers were found to engender clear foreground percepts while colored inducer produced more ambiguous percepts (Guibal and Dresp, 2004). This may be one of the deeper reasons why renaissance painters tended to exploit chiaroscuro and geometric cues to pictorial depth using preferably achromatic contrasts and resorting to color only within a very limited chromatic range. Yet, some differences between colored and achromatic inducers in effects on background brightness and depth seem to shed a new light on this question. These can be are appreciated here by looking at the average data in Figure 9 here (data from analysis 4).

It is shown that the effects of achromatic inducers on figure-ground percepts depend, like the effects of colored inducers, on contrast polarity (ANOVA could not be performed for this comparison), with similar asymmetries between negative and positive polarity ranges. Also, these data suggest some meaningful differences in effects of achromatic and colored inducers with regard to both foreground and background effects. Two additional *post-hoc* comparisons (*t*-tests on individual data, *N* = 2 × 10 for each paired comparison) signaled a significant difference between achromatic and weakly saturated chromatic inducers in their influence on background effects (upper panel in Figure 9) in the positive-to-strong positive polarity range [*t*_(1, 18)_ = 5.44; *p* < 0.001], and between achromatic and fully saturated chromatic inducers in their influence on foreground effects (lower panel in Figure 9) in the positive-to-strong-positive polarity range [*t*_(1, 18)_ = 4.38; *p* < 0.001]. It is therefore likely that weakly saturated chromatic inducers generate more powerful background effects than achromatic inducers, and that fully saturated inducers generate more powerful foreground effects than achromatic inducers within the medium-to-strong-positive polarity range. These results encourage to pursue testing for such differences with a wider range of chromatic achromatic contrasts in different polarity ranges.

The results from this study suggest that induction polarity (assimilation vs. contrast) and depth order (foreground vs. background) cannot be linked by any straightforward causal explanation. While color saturation systematically and significantly determines depth order, this is not so for the case of background brightness induction in terms of contrast or assimilation. Also, one cannot conclude that variables which support contrast systematically bring a contrasted surface to the foreground. In the case of colored inducers, it all depends on their saturation and contrast polarity, and in the case of achromatic inducers, on their contrast polarity. This is consistent with conclusions from earlier studies (e.g., Egusa, 1977, 1983; Guibal and Dresp, 2004; Dresp-Langley and Reeves, 2012) and contradicts the intuition that perceived pictorial depth may be directly linked to subjective brightness effects and color appearance (e.g., Katz, 1911; Long and Purves, 2003). In a review chapter, we (Dresp-Langley and Reeves, 2013) discussed the possibility that a probability based selection of neural signals may drive perceptual grouping (see also Grossberg, 1994; Dresp and Langley, 2005), or Gestalt formation (Dresp, 1997; Pinna, 2011, 2012), and guide the brain in working out the most likely hypothesis of visual structure from elementary characteristics of current visual input.

At some stage, bottom-up attention becomes critically important as some input characteristics readily attract attention away from others in the visual field. Image parts with a stronger and more salient contrast or color may benefit from selection for attention when presented together with objects of a less salient contrast or color. Color saturation may have a decisive influence here. Data from recent visual studies indeed suggest that feature-based selection for attention can be based on any aspect of color contrast. Hue alone may be used independently of lightness in displays with multiple colors, and saturation may be used in displays where color is held constant (Stuart et al., 2014), as was the case in our displays here.

## Conclusion

We conclude that color saturation modulates figure-ground organization, both directly by determining relative inducer depth, and indirectly, and in interaction with contrast polarity by affecting apparent background brightness. The results point toward a hitherto undocumented functional role of color saturation in the genesis of form, and in particular figure-ground percepts. They cannot be accounted for in terms of chromatostereopsis, or summative effects of luminance contrasts. Some interesting differences between effects of achromatic inducers and effects of chromatic inducers highlighted herein deserve further attention. Brightness induction effects (assimilation vs. contrast) and the depth order (foreground vs. background) of surfaces in complex image patterns cannot be linked by any straightforward causal explanation.

### Conflict of interest statement

The authors declare that the research was conducted in the absence of any commercial or financial relationships that could be construed as a potential conflict of interest.
